# Measuring biopsychosocial risk for back pain disability in chiropractic patients using the STarT back screening tool: a cross-sectional survey

**DOI:** 10.1186/s12998-018-0228-5

**Published:** 2019-01-15

**Authors:** Yasmeen Khan, Dana Lawrence, Robert Vining, Dustin Derby

**Affiliations:** 1 Parker University, 2500 Walnut Hill Lane, Dallas, TX 75229 USA; 2 Palmer Center for Chiropractic Research, 741 Brady Street, Davenport, IA 52803 USA; 3 Palmer College of Chiropractic, 1000 Brady Street, Davenport, IA 52803 USA

**Keywords:** STarT Back screening tool, Back pain, Chiropractic, Disability, Back pain disability, Chronic pain, Low Back pain, Biopsychosocial

## Abstract

**Background:**

The Keele STarT Back Screening Tool (SBT), a 9-item questionnaire, screens for pain, physical functioning, fear-avoidance beliefs, catastrophizing, anxious thoughts, low mood, and bothersomeness in persons with back pain. SBT scores designate low, medium, or high risk for developing persistent disabling back pain. The primary study aim was to report the prevalence of SBT-calculated risk for back pain disability in US patients seeking chiropractic care.

**Methods:**

The SBT questionnaire was administered to patients ≥18 years in 3 Chiropractic College outpatient teaching clinics in Iowa and Illinois (May 2017). Descriptive statistics were used to analyze respondent characteristics and prevalence of SBT-calculated risk subgroups. Binary logistic regression analysis was used to examine the relationship between respondent characteristics and SBT scores (including psychological subscores).

**Results:**

Of 550 respondents, 496 completed the SBT; 392 (79%) scored low-risk, 81 (16%) medium-risk, and 23 (5%) high-risk. Mean (SD) age was 44.8 (15.9), 56.9% were female, 88.2% white, 62.6% employed, mean current pain was 2.9 (2.1) out of 10, and 62% reported symptom duration > 3 months. Eighteen percent of respondents reported anxious thoughts, 32% low mood, 41% ≥ 1 and 21% ≥ 3 SBT psychological risk factors. Respondents reporting higher average pain (OR = 1.8 [1.4, 2.3]) and pain severity (OR = 1.3 [1.0 to 1.6]) were more likely to score with medium or high risk. Respondents reporting mid back versus low back pain (OR = 0.2 [0.1, 0.7]), and those employed less than full-time versus full-time (0.2 [01, 0.5]) were less likely to score with medium or high risk. Respondents reporting higher average pain were more likely to report ≥1 psychological factor (OR = 1.8 [1.5, 2.0]). Respondents employed part-time were less likely to report ≥1 psychological factor than those employed full-time (OR = 0.4 [0.2, 0.7]).

**Conclusion:**

The sample surveyed was less likely to score with medium or high risk for back pain disability than previous samples studied, perhaps due to differences in study design and sample characteristics. Rates of low mood and anxious thoughts indicate a need for future research to explore psychological factors among persons seeking chiropractic care.

## Background

Spinal pain is one of the most costly and prevalent conditions worldwide [1–4]. Because of the interplay between musculoskeletal and psychological conditions, spinal pain—back pain in particular—can be considered a biopsychosocial phenomenon [5–10]. While some evidence suggests that psychological factors do not predict outcomes in chiropractic settings [11], anxiety, depression, fear-avoidance behavior, low expectations of recovery, and catastrophizing have been shown to predict poor outcomes in medical and chiropractic settings [6, 7, 12–21]. Furthermore, psychological factors are associated with the transition from acute to chronic back pain [6, 12, 22]. Identifying both biomedical and psychological factors associated with spinal pain facilitates a more comprehensive understanding of patients’ clinical needs [23, 24].

The STarT Back Screening Tool (SBT) is a 9-item questionnaire that screens for physical and psychological risk factors for back pain disability [25, 26]. The SBT was developed within a cohort of patients seeking primary medical care for low back pain (LBP) of any duration (acute, subacute and chronic), with or without referred pain to the lower extremity [25]. Data obtained from the SBT have been studied in several patient populations and exhibit reliability in detecting biopsychosocial risk factors for back pain disability (pain severity, physical functioning, and psychological factors) [20, 27, 28]. More specifically, SBT items inquire about presence of neck or shoulder pain, pain referral to the leg, pain bothersomeness, physical functioning (dressing and walking), fear-avoidance beliefs, catastrophizing, low mood (loss of interest or pleasure), and anxious thoughts [29, 30]. Scores—ranging from 0 to 9—categorize patients with low, medium or high risk for developing back pain disability [29]. The purpose of using the SBT in clinical settings is to screen for risk of developing persistent disabling pain among persons experiencing an episode of LBP. The SBT also identifies patient risk subgroups for targeted treatments (Subgroups for Targeted Treatment, or STarT), meaning that specific treatment plans are matched to SBT score category [29].

Studies report the prevalence of SBT risk subgroups among patients seeking chiropractic care outside of the US [31–35], but similar analyses in US chiropractic patient populations have not been reported. In previous studies, the SBT was shown to be feasibly incorporated into Danish private practice chiropractic clinics [32] and exhibited construct validity in the European populations studied [31, 36]. One study indicated that SBT scores are less predictive among persons with an episode duration under 2 weeks, a characteristic more common among patients seeking chiropractic versus medical care [35]. Another study demonstrated that SBT scores were labile within the first few days after an initial chiropractic visit, also affecting its predictive utility [34]. The wide variety of respondent characteristics and SBT risk profiles among cohorts studied suggests a need for further inquiry.

Nearly 34 million people (14% of the population) in the US sought chiropractic care in 2015 alone [37]. Yet SBT score category prevalence and SBT-informed psychological risk profiles among US patients seeking chiropractic care is still unclear. Therefore, the primary purpose of this study is to report the prevalence of low, medium, and high-risk for back pain disability in adult patients receiving chiropractic care in the US, measured by the SBT. The secondary purpose is to examine the relationship between respondent characteristics and SBT scores (including responses to SBT psychological distress items).

## Methods

### Study design and setting

This study used a cross-sectional survey design. The Palmer College of Chiropractic Institutional Review Board approved this study and granted “exempt” status in April, 2017. Respondents did not share identifying information on the paper surveys. The paper survey was administered at 4 Palmer College of Chiropractic outpatient clinics in 3 locations in Moline, IL and Davenport, IA, US, from May 1–31, 2017. Clinic staff provided eligible participants with a paper copy of the survey as they presented for scheduled office visits. Surveys contained written directions for completion and were comprised of the 9-item SBT and 15 demographic questions to identify sample characteristics. Respondents completed surveys independently and returned them to the front desk staff upon completion.

### Participants and recruitment

Eligible participants were 18 years or older, receiving care at one of the outpatient clinics, and were willing to complete the survey. Participants were instructed to complete the survey only once. To maximize response rate and mitigate coverage error, a study team member (YK) trained office staff to administer surveys as patients presented to the clinics and to make every attempt to supply all eligible patients with a survey. The study team member visited each clinic 1–3 times per week to confirm adherence to study protocols, collect completed surveys, and address questions or concerns.

In line with study aims, any adult presenting to participating clinics during the data collection time frame was permitted to complete the survey, regardless of previous duration of chiropractic care or primary symptom. Although the SBT was designed to be implemented during an acute LBP episode [30], SBT items identify physical and psychological factors that are also clinically pertinent in the management of chronic LBP [38]. In addition, the SBT includes items relevant to both neck and back pain (pain in the shoulder or neck and referred pain to the leg). By including participants of any care duration or symptom region, data collected in the present study reflect the clinical characteristics of a broad range of patients receiving care at US chiropractic teaching clinics.

### Statistical analysis

The primary outcome of interest was the prevalence of each of the 3 SBT risk subgroups. Secondary outcomes included the prevalence of positive responses to each SBT item, the odds of achieving a high-risk score, the odds of achieving a medium or high-risk score (MHRS), and the odds of reporting ≥1 SBT psychological risk factor. The initial study protocol included an analysis of the odds of scoring with high-risk rather than a collapsed medium- and high-risk subgroup. However, the study sample did not contain a large enough pool of respondents with high-risk scores to produce a statistically valid analysis [39].

By collapsing the medium- and high-risk subgroups, the sample size requirement was met, while maintaining clinically relevant results. For example, according to the SBT matched treatment protocol, persons who score with low risk do not receive any further care, while those scoring with medium or high risk are referred for additional treatment. Understanding which patient characteristics may be related to the need for further care is clinically pertinent. The logistic regression analysis of the medium- and high-risk subgroup outcome variable was expected to provide clinical insight and expand the research base related to caring for patients at risk for future disabling pain.

The rationale for exploring factors related to reporting at least 1 psychological factor was similar. For example, previous studies suggest that individual psychological factors alone may contribute to risk for chronic back pain [6, 14]. Because the presence of individual psychological risk factors may affect treatment decisions, the prevalence and likelihood of reporting at least 1 factor is clinically relevant. In line with the study aims, both logistic regression analyses were expected to provide a better understanding of the characteristics of patients receiving chiropractic care in the US and provide a foundation for future research.

We used a double-key entry process to verify data. Analysis of datasets utilized Statistical Package for the Social Sciences (SPSS) version 23.0 (IBM, Chicago, IL). Descriptive statistics using counts and percentages were generated to report demographic characteristics of the total sample, across SBT risk subgroups, and for each of the 9 SBT items.

We performed 3 binary logistic regression analyses. Respondent SBT scores were dichotomized into those who: 1) scored with high risk versus those who did not; 2) those who scored with medium-to-high risk versus those who did not; and 3) those who reported ≥1 psychological risk factor versus those who did not. Exploratory data analysis tested for bivariate interactions between continuous and categorical explanatory variables (Table 1 lists the 13 explanatory variables used in these analyses). Logistic regression model assumptions (a dichotomized outcome variable and independent error terms) were validated. Observations with missing values were not included in this data analysis.

**Table 1:** Descriptive analysis of demographic variables for whole study sample and across STarT Back Tool risk subgroups for patients presenting for chiropractic care (*n* = 550)

Variables	n for whole sample	Whole Sample	n for select variable across STarT Back subgroup	STarT Back Risk Subgroup		
				Low	Medium	High
Age-mean (SD)	529	44.8 (15.9)		42.4 (15.4)	50.9 (17.1)	45.1 (13.6)
Sex-n (%)	536		486			
Male		230 (42.9)		166 (43.2)	33 (41.3)	11 (50.0)
Female		305 (56.9)		217 (56.5)	47 (58.8)	11 (50
Prefer not to answer		0 (0.0)		0 (0.0	0 (0.0)	0 (0.0)
Prefer to self-describe		1 (0.2)		1 (0.3)	0 (0.0)	0 (0.0)
Race-n (%)	524		475			
Native American or Alaskan Native		2 (0.4)		1 (0.3)	1 (0.3)	0 (0.0)
Asian		6 (1.1)		3 (0.8)	2 (2.5)	0 (0.0)
Black or African American		27 (5.2)		13 (3.5)	6 (7.5)	4 (18.2)
Native Hawaiian or Pacific Islander		1 (0.2)		1 (0.3)	0 (0.0)	0 (0.0)
White		462 (88.2)		337 (90.3)	67 (83.8)	16 (72.7)
> 1 race		26 (5.0)		18 (4.8)	4 (5.0)	2 (9.1)
Ethnicity-n (%)	498		453			
Hispanic/Latino		33 (6.6)		23 (6.4)	6 (8.0)	2 (10.5)
Non-Hispanic/non-Latino		465 (93.4)		336 (93.6)	69 (92.0)	17 (89.5)
Employment status-n (%)	511		468			
Full-time		251 (49.1)		201 (54.0)	23 (31.1)	11 (50.0)
Part-time		69 (13.5)		54 (14.5)	9 (12.2)	1 (4.5)
Full-time student		64 (11.6)		53 (14.2)	5 (6.8)	1 (4.5)
Unemployed		127 (24.9)		64 (17.2)	37 (50)	9 (40.9)
Occupation-n (%)	514		467			
Educator		40 (7.8)		36 (9.7)	2 (2.7)	0 (0.0)
Health care professional		61 (11.9)		48 (12.9)	6 (8.1)	1 (0.0)
Active duty military		36 (7.0)		29 (7.8)	4 (5.4)	0 (0.0)
Law enforcement		2 (0.4)		0 (0.0)	1 (1.4)	1 (4.5)
Computer technology		16 (3.1)		12 (3.2)	1 (1.4)	0 (0.0)
Transportation industry		9 (1.8)		8 (2.2)	1 (1.4)	0 (0.0)
Agriculture		10 (1.9)		5 (1.3)	2 (2.7)	0 (0.0)
Other		340 (66.1)		233 (62.8)	57 (77.0)	20 (90.9)
Marital status-n (%)	534		484			
Never been married		147 (27.5)		106 (27.7)	19 (24.1)	6 (27.3)
Married/living with significant other		294 (55.1)		221 (57.7)	45 (57.0)	6 (27.3)
Divorced		75 (14.0)		48 (12.5)	11 (13.9)	8 (36.4)
Widow		18 (3.4)		8 (2.1)	4 (5.1)	2 (9.1)
Primary symptom area-n (%)	549		495			
Low back pain		136 (24.8)		89 (22.8)	30 (37.0)	8 (34.8)
Neck pain		80 (14.6)		66 (16.9)	4 (4.9)	2 (8.7)
Mid-back pain		52 (9.5)		42 (10.7)	4 (4.9)	2 (8.7)
Other		132 (24.0)		109 (27.9)	10 (12.3)	0 (0.0)
> 1 region		149 (27.1)		85 (21.7)	33 (40.7)	11 (47.8)
Current episode duration-n (%)	548		494			
n/a		46 (8.4)		38 (9.7)	2 (2.5)	0 (0.0)
< 2 weeks		60 (10.9)		46 (11.8)	7 (8.8)	4 (17.4)
2–4 weeks		43 (7.8)		32 (8.2)	3 (3.8)	2 (8.7)
5–12 weeks		59 (10.8)		40 (10.2)	10 (12.5)	2 (8.7)
13 weeks – 1 year		71 (13.0)		52 (13.3)	12 (15.0)	2 (8.7)
> 1 year		269 (49.1)		183 (46.8)	46 (57.5)	13 (56.5)
Chiropractic care duration-n (%)	547		493			
n/a or have not received chiropractic care for condition		52 (9.4)		39 (7.2)	4 (4.9)	1 (4.3)
< 2 weeks		41 (7.5)		29 (7.5)	5 (6.2)	2 (8.7)
2–4 weeks		49 (9.0)		34 (8.7)	9 (11.1)	1 (4.3)
5 to 12 weeks		67 (12.2)		50 (12.9)	10 (12.3)	2 (8.7)
13 weeks – 1 year		78 (14.3)		58 (14.9)	9 (11.1)	4 (17.4)
> 1 year		260 (47.3)		179 (46.0)	44 (54.3)	13 (56.5)
Average pain in past 2 weeks-mean (SD)	547	3.6 (2.2)		3.0 (1.8)	5.5 (1.8)	7.3 (1.5)
Most severe pain in past 2 weeks-mean (SD)	547	5.1 (2.6)		4.4 (2.4)	7.2 (1.8)	8.7 (1.3)
Current pain-mean (SD)	530	2.9 (2.1)		2.3 (1.8)	4.6 (1.8)	5.9 (2.6)

We used a simple binary logistic regression analysis to compute odds ratios (ORs) and 95% confidence intervals for the likelihood of achieving a high-risk score, a medium-to-high-risk score, and reporting ≥1 psychological risk factor, based on each explanatory variable.

We considered individual explanatory variables and interactions that produced statistically significant ORs (*p* ≤ 0.1) for inclusion in multiple logistic regression analyses. All variables that had a statistically significant (*p* ≤ 0.05) contribution to the odds for each outcome variable were considered for inclusion in the final models.

## Results

Of the 599 eligible patients presenting in May, 2017, 550 completed surveys, resulting in a 91.8% response rate. Four hundred ninety-six respondents completed all 9 SBT items (response rate = 90% of respondents). Table 1 summarizes the study population’s baseline demographic and clinical characteristics. The mean (SD) age of the sample was 44.8 (15.9) and the mean current pain reported on a 0–10 scale was 2.9 (2.1). The majority of the sample was female (57%), Caucasian (88%), and employed part- or full-time (63%). Twenty-five percent of respondents selected a primary symptom of LBP, 15% neck pain, and 10% mid back pain, 27% more than 1 pain region, and 24% “other” indicating a reason for presenting to the clinic that was not listed. The majority (62%) of respondents reported an episode duration over 12 weeks. Most (62%) also reported having received chiropractic care for over 12 weeks for their presenting condition.

### STarT Back subgroup and item prevalence

The primary aim of this study was to report the prevalence of each SBT risk subgroup. Of the 496 respondents who completed all SBT items, 392 (79.0%) achieved a low-risk SBT score, 81 (16.3%) a medium-risk score, and 23 (4.6%) a high-risk score. Among persons reporting a primary symptom of LBP (*n* = 127), 89 (70%) scored with low risk, 30 (24%) scored with medium risk, and 8 (6%) scored with high risk. Among persons with an episode duration under 2 weeks (*n* = 57), 46 (81%) scored with low risk, 7 (12%) with medium risk, and 4 (7%) with high risk.

Secondary aims were to report the mean SBT score and the proportion of positive responses to each SBT item. Table 2 summarizes the descriptive statistics for SBT total and item scores. The mean (SD) SBT total score was 2.1 (1.9). Of respondents completing the SBT, 32% reported low mood (loss of interest), 18% reported anxious thoughts, 17% reported ‘very much’ or ‘extremely’ bothersome pain, 11% reported catastrophic thoughts, and 9% reported fear-avoidance beliefs.

**Table 2:** Mean STarT Back scores and descriptive characteristics by domain

STarT Back total score-mean (SD)	*n* = 496	2.1 (1.9)
STarT Back-5 psychological distress subscore-mean (SD)	*n* = 506	0.8 (1.2)
Referred pain to leg (Y)-n (%)	*n* = 539	94 (17.4)
Shoulder or neck pain (Y)-n (%)	*n* = 544	412 (75.7)
Physical Function: Only walked short distances (Y)-n (%)	*n* = 537	99 (18.4)
Physical Function: Dressed more slowly (Y)-n (%)	n = 537	100 (18.6)
Fear-avoidance: Not safe to be active (Y)-n (%)	*n* = 532	48 (8.7)
Anxiety: Worrying thoughts (Y)-n (%)	*n* = 536	95 (17.7)
Catastrophizing: Back pain is terrible and never going to get better (Y)-n (%)	n = 537	57 (10.6)
Depression: Not enjoying things I used to (Y)-n (%)	*n* = 531	168 (31.6)
Bothersomeness (very much to extremely)-n (%)	*n* = 538	91 (16.5)

The Odds of Scoring in a Medium-to-High-Risk Group and Reporting Psychological Risk Factors.

Another secondary aim was to report which demographic and clinical characteristics contributed to the odds of scoring in the high-risk group, the medium-to-high-risk group, and reporting ≥1 psychological risk factor. However, we did not obtain a large enough pool of high-risk respondents to conduct a sound regression analysis, hence the post hoc change to a pooled medium-to-high risk group. Tables 3 and 4 report ORs and 95% confidence intervals for the odds of achieving a MHRS and reporting ≥1 psychological risk factor.

**Table 3:** Odds ratios for scoring in the medium or high-risk STarT Back subgroups based on select explanatory variables^a^

Explanatory Variable	OR	95% Confidence Interval	*p*-value
Average pain in past 2 weeks	1.8^c^	(1.4, 2.3)	< 0.001
Most severe pain in past 2 weeks	1.3^c^	(1.0, 1.6)	0.02
Primary symptom area			
Low back pain^b^	–	–	
Neck pain	1.0	(0.5, 2.1)	1.00
Mid-back	0.2^c^	(0.1, 0.7)	0.02
> 1 area of complaint	0.4	(0.1, 1.1)	0.11
Other	0.3	(0.1, 1.3)	0.07
Employment status			
Full-time^b^	–	–	
Part-time	0.2^c^	(0.1, 0.5)	< 0.001
Full-time student	0.3^c^	(0.1, 0.7)	0.01
Unemployed	0.0^c^	(0.1, 0.7)	0.02

^a^Hosmer and Lemeshow Test *p*-value = 0.799

^b^Referent category

^c^indicates statistical significance at alpha = 0.05 level

**Table 4:** Odds of reporting ≥1 psychological risk factor based on select explanatory variables^a^

Explanatory Variable	OR	95% Confidence Interval	*p*-value
Average pain in past 2 weeks	1.8^c^	(1.5, 2.0)	< 0.001
Employment status			
Full-time^b^	–	–	
Part-time	0.4^c^	(0.2, 0.7)	0.001
Full-time student	0.5	(0.2, 1.0)	0.05
Unemployed	0.8	(0.4, 1.9)	0.62

^a^Hosmer and Lemeshow Test p-value = 0.950

^b^Referent category

^c^indicates statistical significance at alpha = 0.05 level

One hundred and four (20.9%) respondents were categorized with MHRSs. Average pain rating in the past 2 weeks (on a 0 to 10 scale), most severe pain rating the past 2 weeks (on a 0 to 10 scale), primary symptom, and employment status correctly predicted a MHRS 89% of the time. Respondents reporting higher average pain intensity were more likely to achieve a MHRS (OR = 1.8 [1.4, 2.3, *p* <  0.001; Table 3). Respondents reporting higher rates of the most severe pain intensity in the past 2 weeks were more likely to achieve a MHRS (OR = 1.3 [1.0, 1.6], *p* = 0.02).

Respondents reporting neck pain as a primary symptom reported approximately the same likelihood of achieving a MHRS compared to those reporting LBP. Those reporting LBP were more likely to achieve a MHRS than those reporting mid back pain, more than 1 primary symptom category, and those who answered “other.” However, mid back pain was the only symptom category with a statistically significant effect on the odds of a MHRS, relative to the LBP referent category (OR = 0.2 [0.1, 0.7], *p* = 0.02). In other words, reporting a primary symptom of low back pain instead of mid back pain was associated with an increased odds of achieving a MHRS.

Respondents who were employed part-time, were full-time students, or were unemployed were less likely to achieve a MHRS compared to those who were employed full-time (OR = 0.2 [0.1, 0.5], *p* <  0.001; OR = 0.3 [0.1, 0.7], *p* = 0.01; OR = 0.0 [0.1, 0.7], *p* = 0.02). In other words, full-time working status was associated with increased odds of achieving a MHRS.

Forty-one percent of respondents reported ≥1 SBT-identified psychological risk factor, with low mood reported most commonly. Average pain intensity in the past 2 weeks and employment status correctly predicted the presence of ≥1 psychological condition 74% of the time. Respondents with higher average pain intensity were significantly more likely to report ≥1 psychological risk factor (OR = 1.8 [1.5, 2.0], *p* <  0.001); Table 4). Respondents who were part-time employed, full-time students, or unemployed were less likely to report ≥1 psychological risk factor compared to those were employed full-time. However, part-time employment status was the only employment category that had a statistically significant effect on the odds of reporting ≥1 psychological risk factor, relative to the full-time status referent category (OR = 0.4 [0.2, 0.7], *p* = 0.001).

## Discussion

To our knowledge, this is the first study to report the prevalence of SBT-designated risk subgroups among persons in the US seeking chiropractic care. Compared with chiropractic patient cohorts in previous studies, respondents in this study achieved MHRSs at lower rates [31–34]. Most previously published studies employing the SBT in chiropractic settings were conducted within patient cohorts reporting an acute episode of non-specific LBP [31, 32, 34] whereas the present study allowed inclusion of all adult patients regardless of episode duration or primary symptom. Overall, 91% of respondents had received some form of chiropractic care for their condition at the time of data collection, and less than half reported a primary symptom of LBP. Nevertheless, among respondents with an episode duration under 2 weeks, a chiropractic care duration under 2 weeks, and LBP, the prevalence of MHRSs was lower than rates reported in previous chiropractic cohorts [31–35]. This indicates that these factors alone cannot explain the lower rates of MHRSs in the present study.

In the present study, care duration was not significantly associated with a MHRS. On the other hand, reporting low back relative to mid back pain was associated with an increased likelihood of a MHRS. Furthermore, among respondents reporting a primary symptom of LBP (versus neck or mid back pain), the SBT score distribution more closely matched distributions reported in previous chiropractic settings (Present study, primary symptom LBP: low = 70%, medium = 24%, high = 6%; Kongsted et al., 2011: low = 59%, medium = 29%, high = 11%; Morso et al., 2016: low = 52%, medium = 39%, high = 10%) [32, 35, 40]. Similarly, among respondents reporting more than 1 region of pain (versus neck or mid back pain), SBT score distributions more closely matched those reported in previous studies (low = 66%, medium = 26%, high = 9%; Table 1). However, among respondents reporting LBP or more than 1 symptom, and within the overall sample, the prevalence of MHRSs was lower than in previous chiropractic patient cohorts [40]. Based on the study design and data collected, it is unclear how differences between the current study sample and those described in previous studies may have contributed to the lower prevalence of MHRSs in this sample.

Despite a lower prevalence of MHRSs, responses indicating low mood, anxious thoughts, catastrophizing, and pain bothersomeness were common (Table 2). For example, 32% of respondents reported low mood (loss of interest or pleasure), 19% reported anxious thoughts, 16% reported catastrophic thoughts, and 22% reported a perception that their pain was ‘very much’ or ‘extremely’ bothersome. Although individual SBT items are not diagnostic of mental health conditions, 19% of respondents reported having anxious thoughts, consistent with prevalence rates for anxiety disorders among US adults (19%) [41]. On the other hand, 32% of respondents reported low mood (loss of interest or pleasure) compared to 6.7% of the US adult population reporting 1 or more depressive episodes in a given year [42]. A loss of pleasure may be more common than clinical depression itself, however data collected in this study cannot explain the high rates of low mood among respondents.

Results of this study indicate that respondent pain severity was associated with reporting 1 or more SBT psychological risk factors. Average pain in the past 2 weeks was significantly associated with increased odds of reporting ≥1 SBT psychological risk factor, and achieving a MHRS. This result confirms previous findings suggesting that higher pain scores are associated with higher SBT total and SBT-5 psychological distress subscores [35, 43, 44].

The relationship between full-time employment status and SBT risk category is also notable and worth future scientific investigation. There may be a relationship between specific vocational activities and SBT score over time. Because a substantial proportion of respondents had received prior chiropractic care, results of the logistic regression analysis may not indicate whether employment status, pain severity, and pain location are related to an initial high risk for persistent back pain disability. Instead, these analyses may indicate which demographic factors are associated with a change in SBT scores over time, or the maintenance of relatively high scores over time. It may be noted that the majority of respondents scoring with high risk (57%) reported receiving care for more than 1 year (Table 1). The analyses conducted for the present study cannot explain the association between full-time employment status, pain severity or location and SBT item responses.

This study has several limitations inherent to its design. Due to the observational study design, causative factors influencing SBT scores cannot be identified, and study results must be interpreted conservatively. Also, there may have been differences in SBT scores and demographic characteristics between respondents and non-respondents, leading to non-response error. The high response rate (92% overall, 83% completing the entire SBT) mitigates this potential to a large extent. Next, despite attempts to minimize measurement error, patient self-reporting can limit data accuracy and validity. Coverage error was possible because the survey was administered at clinics in 1 geographic location. Therefore, study results may not be generalizable to all US patients seeking chiropractic care.

The SBT is a screening instrument designed to be employed at the onset of care for a LBP episode. Yet, most respondents in the present study had already received some form of chiropractic care for their condition at the time of data collection. Because previous literature demonstrates that SBT scores are labile both in the initial stages and over the course of chiropractic care [31, 34], the greater prevalence of persons having received chiropractic care may have contributed to the lower rates of MHRSs relative to previous studies in chiropractic settings. Similarly, less than half of respondents reported a primary symptom of LBP. As a result, the SBT subgroup classifications may not be predictive of outcomes in some respondents. Analyses conducted for the present study cannot explain the lower prevalence of MHRSs relative to previous studies conducted in chiropractic settings [31–35], although respondents reporting LBP were more likely to score with medium or high risk. Because there are few studies of this kind, similar research efforts focused on US private practice settings could provide additional insight into the characteristics of a broad range of chiropractic patients.

### Clinical implications

Results suggest that a clinician in the study setting might expect 1 out of every 2 to 3 patients to be at risk for depression, 1 out 4 to 5 to be at risk for anxiety, and approximately 1 out of 6 patients to be at risk for potentially catastrophizing thought patterns that may impede patient progress. Doctors of chiropractic, like many other primary contact healthcare providers, may consider augmenting their knowledge about, and preparedness for, managing care for patients experiencing fear-avoidance beliefs, catastrophizing, depression, anxiety, and perceived pain bothersomeness [45–47].

Previous trials examining the use of STarT Back matched treatment plans suggest that healthcare providers who work with back pain-related conditions can improve patient outcomes by implementing cognitive behavioral approaches to assist in overcoming psychological barriers to recovery [29, 48]. Some chiropractic clinicians currently implement strategies to help patients overcome maladaptive cognitive and emotional responses to pain, such as graded exposure and behavioral interventions [49]. The prevalence of psychological risk factors among respondents in this study suggests the importance of clinician awareness of the common need for such interventions.

## Conclusion

Seventy-nine percent of respondents scored with low risk for developing back pain disability, 21% scored medium-to-high risk for developing back pain disability, and 41% reported at least 1 SBT psychological risk factor. The SBT is a screening tool and cannot be used to diagnose mental health conditions. However, a relatively high prevalence of respondents indicating low mood and anxious thoughts supports the need for future research efforts aimed at quantifying the prevalence of common psychological risk factors and mental health conditions among US patients seeking chiropractic care. Survey results suggest that clinicians at US chiropractic teaching clinics should develop, or continue implementing, management pathways for patients experiencing specific types of psychological distress. Future studies examining how SBT-informed tailored treatment plans influence pain, disability, and quality of life outcomes are also needed.

## Abbreviations

LBP: Low back pain
MHRS: Medium-to-high-risk score
SBT: STarT Back Screening Tool
